# Mantle redox state drives outgassing chemistry and atmospheric composition of rocky planets

**DOI:** 10.1038/s41598-020-67751-7

**Published:** 2020-07-02

**Authors:** G. Ortenzi, L. Noack, F. Sohl, C. M. Guimond, J. L. Grenfell, C. Dorn, J. M. Schmidt, S. Vulpius, N. Katyal, D. Kitzmann, H. Rauer

**Affiliations:** 1 Institute of Planetary Research, German Aerospace Center, Rutherfordstr. 2, 12489 Berlin, Germany; 2 Department of Earth Sciences, Freie Universität Berlin, Malteserstr. 74-100, 12249 Berlin, Germany; 3 Department of Earth Sciences, Bullard Laboratories, University of Cambridge, Madingley Rise, Cambridge, CB3 0EZ UK; 4 Institute of Computational Sciences, University of Zurich, Winterthurerstr. 109, 8057 Zurich, Switzerland; 5 Department of Astronomy and Astrophysics, Technical University of Berlin, Hardenbergstr. 36, 10623 Berlin, Germany; 6 University of Bern, Center for Space and Habitability, Gesellschaftsstr. 6, 3012 Bern, Switzerland

**Keywords:** Planetary science, Astronomy and planetary science

## Abstract

Volcanic degassing of planetary interiors has important implications for their corresponding atmospheres. The oxidation state of rocky interiors affects the volatile partitioning during mantle melting and subsequent volatile speciation near the surface. Here we show that the mantle redox state is central to the chemical composition of atmospheres while factors such as planetary mass, thermal state, and age mainly affect the degassing rate. We further demonstrate that mantle oxygen fugacity has an effect on atmospheric thickness and that volcanic degassing is most efficient for planets between 2 and 4 Earth masses. We show that outgassing of reduced systems is dominated by strongly reduced gases such as $\text {H}_{2}$, with only smaller fractions of moderately reduced/oxidised gases ($\text {CO}$, $\text {H}_{2}\text {O}$). Overall, a reducing scenario leads to a lower atmospheric pressure at the surface and to a larger atmospheric thickness compared to an oxidised system. Atmosphere predictions based on interior redox scenarios can be compared to observations of atmospheres of rocky exoplanets, potentially broadening our knowledge on the diversity of exoplanetary redox states.

## Introduction

Atmospheric evolution on a rocky planet is affected by numerous fundamental processes. Generally, atmospheres can be accreted from the proto-planetary disk (primordial atmosphere), or outgassed from the interior either during the cooling of a magma ocean (primary atmosphere) or by volcanism during the planet’s long-term geological history (secondary atmosphere). The total volume of volcanic outgassing is the sum of the contribution from the deeper magmatic emplacements, also called passive degassing, and from extrusive volcanism (discussed in detail in the supplementary materials). Furthermore, atmospheres can be lost via escape (driven by thermal or non-thermal processes or via large impacts) and enriched via delivery from smaller impacts^1^. The interplay of these central processes determines the atmospheric evolution and observable gas species in the atmosphere. Gaseous species released from present-day volcanoes on Earth are dominated by $\text {H}_{2}\text {O}$ and $\text {CO}_{2}$ which are strong greenhouse gases^2^. Consequently, a planet’s potential to develop habitable conditions is linked to interior processes in a variety of ways^3^.

The oxidation state of the mantle at a given point in time depends on numerous factors such as the primordial disk composition, core-mantle processes and loss of atmospheric hydrogen to name but a few^4^. During the core segregation process, the co-existence of liquid iron and silicate led to a reduced mantle^5^. The oxidation of the mantle is related to core differentiation and depends on factors such as the mantle pressure, temperature and stellar irradiation^6,7^. The Earth’s upper mantle is oxidized at present; geological evidence suggests that this transition from a reduced mantle to an oxidized one occurred during its earliest evolution, although the exact timing is still debated^8–13^. Whether rocky exoplanets are expected to follow an analogous pattern in redox state represents a major uncertainty in the understanding of their atmospheric evolutions. Previous model studies of rocky planets have suggested that the efficiency and the composition of volcanic outgassing depend on properties such as mass, thermal state, age, tectonic style and planetary bulk composition^14–18^. Here, we simultaneously investigate the different phases of the volatile pathways from the interior to the atmosphere, combining processes that are usually analysed separately. We analyse planets operating in a stagnant lid tectonic regime. We do not consider whether any lingering gases from the primary atmosphere would affect the compositions of the secondary atmospheres we model. This assumes that the species degassed during a short magma ocean stage would be lost or replaced within billions of years of volcanic outgassing. A massive atmosphere during the magma ocean phase most likely implies a surface still molten and therefore, not a stagnant lid planet^19,20^. In particular, we analyse how a planet’s mantle redox state affects its outgassed atmospheric composition through the double influences of mantle-melt volatile partitioning and gas chemical speciation. Whether outgassing is dominated by reduced or oxidized species can regulate the atmospheric scale height (Eq. 27) via the mean molecular mass. The smaller molecular weights of $\text {CO}$, $\text {H}_{2}\text {O}$ and $\text {H}_{2}$ as compared to $\text {CO}_{2}$ generate less dense atmospheres leading to a larger atmospheric thickness. In our work, we consider the three main elements on Earth which together constitute the bulk of Earth’s volatile molecular inventory and which are central for determining surface habitability conditions, namely C, H and O.

## Results

To investigate the variations in atmospheric composition and surface pressure for rocky exoplanets of different masses, we consider the influence of the initial volatile content in the magma and the melt redox state. Our numerical model simulates the volatile flux from the mantle to the atmosphere, as detailed in the methods section. In Sect. 4.2 we calculate the volatile solubility in the silicate melt and the gas chemical speciation of the C–O–H system. Finally, in Sect. 4.3, we simulate the atmospheric evolution analysing the final volatile composition and the atmospheric radial extent. Dorn et al.^18^ modelled in total 2,340 different rocky planet evolutionary cases by varying initial conditions and major element compositions of hypothetical exoplanets, while assuming a fixed amount of $\text {CO}_{2}$ melt concentration and oxidised conditions in the melt. Here, we use the same cases as reported in^18^ but consider that both $\text {H}_{2}\text {O}$ and $\text {CO}_{2}$ are stored in the mantle subsequent to the solidification of the magma ocean^21,22^, combined with variable redox scenarios. Table 1 lists different cases of volatile amounts in the melt, based on three volatile delivery cases investigated for Earth in^21^. In our simulations, we employed as starting volatile contents of the mantle the $\text {H}_{2}\text {O}$ and $\text {CO}_{2}$ stored after the magma ocean solidification (Table 1). Whereas the first and second cases assume different relative amounts of $\text {CO}_{2}$ and $\text {H}_{2}\text {O}$, the third case considers a dry scenario where only $\text {CO}_{2}$ was delivered. To investigate the influence of $\text {H}_{2}\text {O}$ degassing for a more carbon-rich mantle, we add a small amount of water to the third case. We consider different mantle redox states reproducing an oxidised (Iron–Wuestite buffer) and a reduced scenario (Quartz–Fayalite–Magnetite buffer) and their influence upon the amount and composition of the outgassed volatiles (see Sect. 4.2). This results in a total of 28,080 evolutionary scenarios.

**Table 1:** Initially delivered volatile concentrations in the magma ocean (weight percent) and resulting volatile fractions stored in the mantle after the magma ocean solidification, based on different initial delivery scenarios from^21^.

Case	Delivered		Stored	
	$\text {H}_{2}\text {O}$	$\text {CO}_{2}$	$\text {H}_{2}\text {O}$	$\text {CO}_{2}$
Low	0.05	0.01	0.015	0.0022
High	0.5	0.1	0.045	0.005
Dry	0	0.6	0.005^a^	0.018

The $\text {CO}_{2}$ is stored as graphite in the mantle. $^{\text {a}}$We consider here a small water fraction instead of the dry case investigated in^21^.

Given the initial volatile contents in the mantle, the corresponding concentrations in the melt are regulated by the partitioning between the mantle and the melt produced. For carbon species, this is directly influenced by the oxidation state of the system (Eq. 2). The main effect is that for reducing conditions, the dissolution of carbonates from the mantle rocks into the melt is suppressed, and therefore the volatile content in the magma will be dominated by water. On the other hand, an oxidising scenario favours an enrichment in carbonates in the melt. Hence, melts with different volatile contents can be generated even if they originate from source rocks with identical starting volatile concentrations but different redox states. Once the melt reaches the surface, we simulate the dissolved gas chemical speciation in the C–O–H system.

**Fig. 1:**
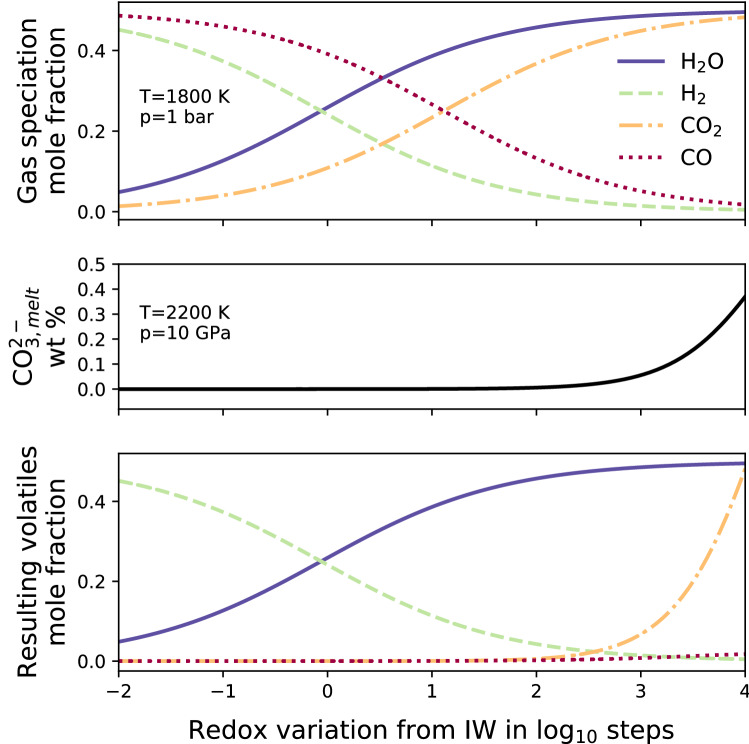
Composition of outgassed volatiles as a function of oxygen fugacity which is shown in logarithmic values relative to the Iron–Wuestite buffer, where the initial volatile composition is 50 mol% for both $\text {H}_{2}\text {O}$ and $\text {CO}_{2}$. The most oxidised case shown here (IW+4) reflects a redox state similar to Earth’s upper mantle at present day temperature. Top: outgassed volatile composition without considering the mantle-melt composition (i.e. starting volatile composition is considered in the melt). Middle: weight percent of carbonates dissolved in the melt as a function of oxygen fugacity at 2200 K and 10 GPa. Bottom: outgassed volatile composition considering the mantle-melt volatile partitioning and the volatile chemical speciation.

Figure 1 illustrates the combined effects of volatile melt partitioning and speciation on the ultimate outgassing chemistry. The upper panel isolates the redox-dependence of the surface (1 bar pressure) gas speciation for a constant volatile concentration in the melt; without calculating the melt partitioning. This essentially reproduces^23^: $\text {H}_{2}$ and $\text {CO}$ are the most abundant outgassed species in the more reducing states (log $f_{O_2} < 0$), while in the more oxidised scenarios (log $f_{O_2} > 1$), the principal gas phases are $\text {H}_{2}\text {O}$ and $\text {CO}_{2}$. However, as described above, the volatile concentrations in the melt depend on redox state as well (middle panel). If both of these effects are coupled (bottom panel), we get rather different results for the outgassed composition. Now in reducing scenarios, $\text {H}_{2}$ is the most outgassed species, while zero-to-little $\text {CO}$ is degassed because the carbonate partitioning in the melt is inhibited. Upon increasing the oxidation state, carbonate starts to become present and the outgassing is dominated by $\text {CO}_{2}$ and $\text {H}_{2}\text {O}$.

**Fig. 2:**
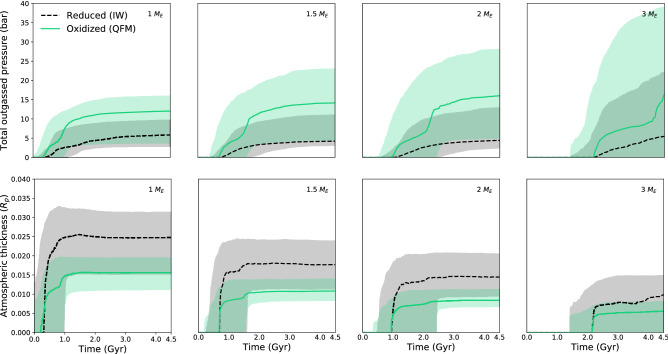
Shaded area is the $1\sigma$ variation while lines represent the median of the modelled evolution of atmospheric thickness bottom panel) and outgassed pressure (top panel) over 4.5 Gyr, where subplots group simulations by planet mass. Black dashed lines represents reduced mantles (IW buffer), while green solid lines represents oxidised cases (QMF buffer).

**Fig. 3:**
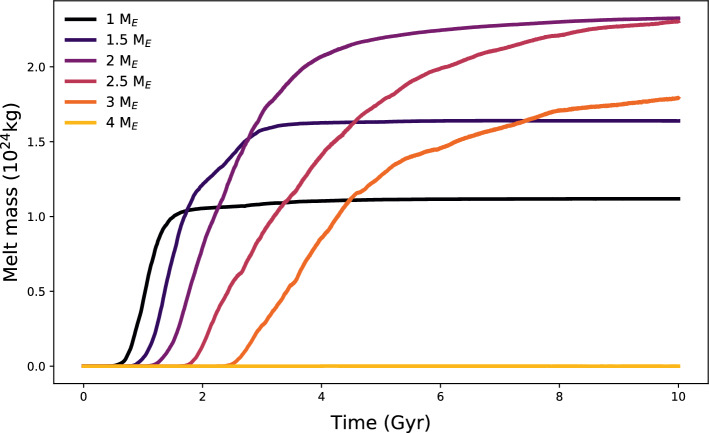
Melt production over time (Gyr) for different planet sizes considering an Earth-like interior structure and composition. With increasing planet mass given in units of Earth masses (*M*_*E*_), there is a delay in the production of melt and no further magma generation for massive planets above 4$M_E$.

**Fig. 4:**
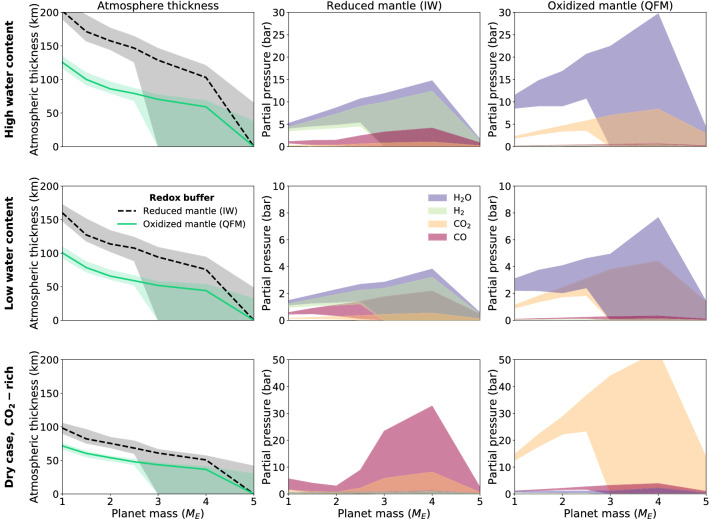
Change of atmospheric thickness and outgassed partial pressures with planetary mass after 4.5 Gyr of simulated mantle convection. Individual panels compare reduced mantles (IW buffer) with oxidised mantles (QFM buffer), and rows indicate different initial volatile cases (i.e. dry, low, high) from Table 1. The left column of the panel shows the mass-dependence of modelled atmospheric thickness, comparing reduced mantles (black dashed line) with oxidised mantles (green solid line). Shaded areas show the 1$\sigma$ variation across all simulations therein, while the lines denote the median. In the central and right column we examine the partial pressures of $\text {H}_{2}\text {O}$ (purple swaths), $\text {H}_{2}$ (green swaths), $\text {CO}_{2}$ (orange swaths), and $\text {CO}$ (red swaths). Shaded areas show the 1$\sigma$ variation across all simulations therein, considering that for a given volatile and redox scenario, factors causing variation in atmospheric thickness include bulk Mg/Fe/Si ratios, initial mantle temperature profiles and heat sources.

Atmospheric evolution is analysed considering melt fluxes over time in combination with the volatile solubility and the chemical equilibrium during the outgassing process. Therefore, the scenarios performed here are affected by the melt production, temperature, pressure, volatile content and oxidation state of the system. For simplicity, we select the atmospheric pressure at the surface as the outgassing pressure. The pressure has a direct influence on the volatile solubility in the melt and the outgassed composition. The bottom panel of Fig. 2 displays the median and 1$\sigma$ variation (68% confidence interval) of modelled atmospheric thickness, expressed in units of planetary radius over time for the four different planetary masses investigated. Results suggest a general decrease in atmospheric thickness with increasing planetary mass, hence increased surface gravity in the scale height definition (see Eq. 27), and larger radial atmospheric extent for reducing conditions. Figure 2 (top panel) shows the atmospheric pressure over time at different redox states and planet sizes. In the analysed cases, highest outgassing values occur for planets of several Earth masses, and oxidised mantles lead to higher atmospheric surface pressure as compared to reduced mantles since the oxidised volatile species have a higher molecular weight than reduced gases (in the case of $\text {H}_{2}\text {O}$ versus $\text {H}_{2}$ the weight varies by a factor of nine). After 4.5 Gyr of mantle convection, most degassing from the interior (if it occurred) had already taken place^18^, although in general the more massive the planet, the later degassing occurs. The outgassing lag for increasing planetary mass is due to the larger internal pressure which produces a higher mantle viscosity. The rheology variation reduces the vigor of mantle convection so that outgassing starts later compared to planets with lower mass (Fig. 2). An increasing planet mass leads to higher initial mantle temperatures and an increased inventory of volatiles and radioactive heat sources. This is reflected in a larger amount of volcanic activity and outgassing for planets with masses up to 2–4 $M_E$ (depending on the specific simulation parameters). However, for planets of masses above this threshold, we can observe a negative trend in volcanic activity. This trend is directly related to the increasing planetary mass and surface gravitational acceleration and therefore increasing pressure gradient in the lithosphere. The pressure at the bottom of the lithosphere leads to a higher melting temperature than for Earth-mass planets, causing reduced or even no outgassing (see Fig. 3 using longer, sample evolution scenarios up to 10 Gyr for planets of different masses but with the most Earth-like composition scenario in our data set).

Figure 4 compares atmospheric thickness and the resulting partial pressures obtained for all investigated cases as a function of the planetary mass. The degassing trend still reflects that under oxidising conditions the atmospheric pressure will be larger and mainly composed of $\text {H}_{2}\text {O}$ and $\text {CO}_{2}$. On the other hand, under reducing conditions the atmospheric pressure is lower because a part of the $\text {H}_{2}\text {O}$ content is replaced by $\text {H}_{2}$ and $\text {CO}$ outgassing is favoured over $\text {CO}_{2}$ though strongly limited due to the smaller carbonate content in the reduced melt. Concerning the influence of the planetary mass, for both reducing and oxidising cases, maximum degassing occurs between 2 and 4 Earth masses. A higher molecular weight of the atmosphere leads to a shallower atmospheric vertical extent. This can be seen in the left column of Fig. 4, which shows the median and 1$\sigma$ variation of atmospheric thickness as a function of the planetary mass after 4.5 Gyr of mantle convection, for different initial volatile contents. For all the volatile scenarios, the oxidation level leads to strongest differences in atmospheric thicknesses for the low-mass planets. In the case of a more reduced mantle, the atmospheric thickness is generally larger compared to the oxidised case. However, this difference decreases as the planetary mass increases. In addition, results suggest either low or virtually zero outgassing rates for the more massive planets considered here, consistent with^16,18^.

**Fig. 5:**
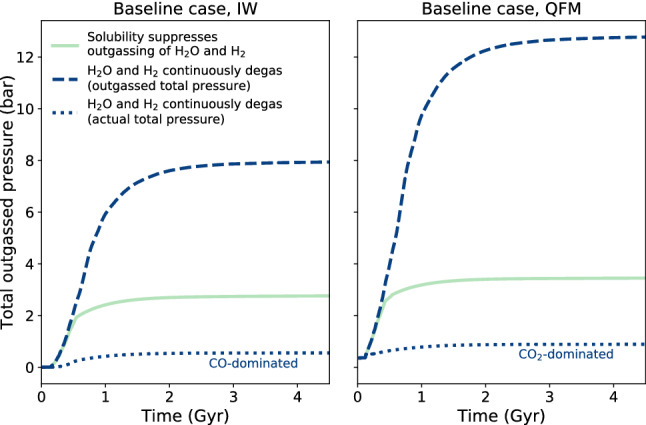
Evolution of an outgassed atmosphere’s total pressure for different scenarios, showing a single case (mass = 1 $M_{\oplus}$, initial volatile concentrations $X_{\text {CO}_{2}}$ = 22 ppm, $X_{\text {H}_{2}\text {O}}$ = 150 ppm, Fe/Si = 0.5, Mg/Si = 1.0) as an example. The dashed blue line represents the atmospheric evolution without considering solubility, hydrogen escape, or water condensation. The solid green line shows the melt solubility effect on outgassing, but neither hydrogen escape nor water condensation are simulated. The dotted blue line shows the case where hydrogen escape and water condensation are simulated.

We performed simulations considering also the $\text {H}_{2}$ atmospheric escape and the $\text {H}_{2}\text {O}$ condensation in an ocean layer. Figure 5 shows surface pressure as a function of time for an Earth mass planet assuming that all the hydrogen is lost to space via escape, and all of the water retained in oceans. These results suggest that the evolution of the atmospheric composition is strongly linked/coupled with the surface pressure. The dashed blue lines denote runs which assume no solubility limitations to the outgassing of $\text {H}_{2}$ and $\text {H}_{2}\text {O}$ and resulting in a large atmospheric pressure at surface. For the reducing state (IW buffer), the surface pressure is lower compared to the oxidised case (QFM buffer), as expected. The green solid lines denote runs which show the effect of solubility without hydrogen escape and water condensation (all gases remain in the atmosphere). Here, due to the solubility effect, there is a slight difference in the surface pressure between the IW and the QFM buffers. The dotted lines represent the scenario where all the hydrogen escapes and the water condenses. In this case, the pressures at the surface are lower compared to the other cases analysed. The chemistry of the atmosphere reflects the reducing or oxidizing nature of the mantle in terms of a $\text {CO}$- versus a $\text {CO}_{2}$-dominated atmosphere.

**Fig. 6:**
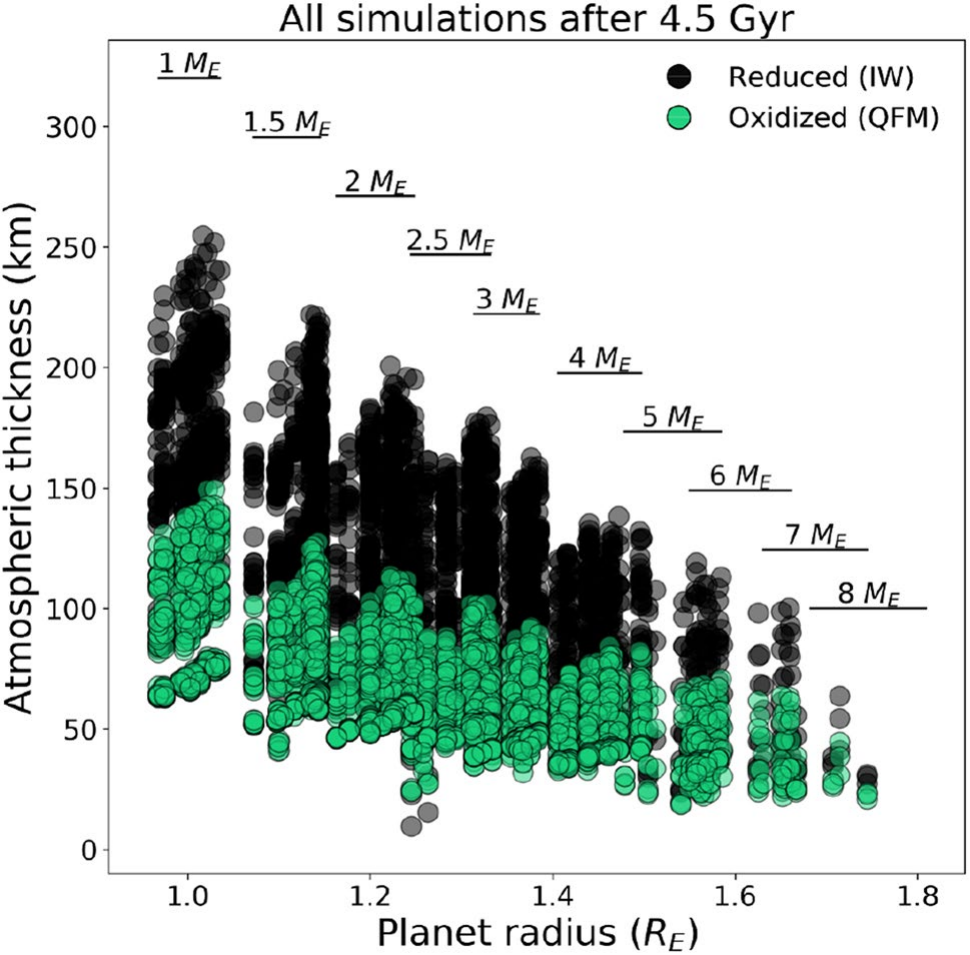
Scatter plot showing calculated atmospheric thicknesses versus planetary radii of all 7,650 scenarios which result in outgassing. Colours indicate mantle redox buffers (black is IW; green is QFM). The range of planetary radii corresponding to the individual input planetary masses are marked with horizontal lines.

To summarise the effect of the oxygen fugacity on the radial extent of the atmosphere, Fig. 6 compares the calculated atmospheric thicknesses for different planet radii considering the two different petrological mineral buffers assuming no atmosphere losses. There is a marked difference between the atmospheric thickness for the reducing (IW) compared to the oxidising (QFM) scenario. In all the cases analysed, the reduced states have larger atmospheric thicknesses than the oxidised scenarios. It is interesting to note, that even though the reducing scenario atmospheres have smaller masses and densities, in our simulations their atmospheric thicknesses are larger than in the oxidised cases. This is due to the different molecular weights of the outgassed species. In a reducing scenario the volatiles have a smaller molecular weight and this results in a larger atmospheric thickness.

## Discussion

Our numerical simulations show that the mantle’s redox state influences the volatile content of the melt as well as the volatile chemistry during degassing. On the other hand, planetary mass affects the total volume of melt that is produced and hence the volatile depletion of the mantle. Some authors^24,25^ have suggested there is an influence of planet mass on the oxidation of the mantle. More massive planets are able to reach higher central pressures, which facilitate more effective disproportionation of ferrous iron and its segregation into the core. Further^26^, have hypothesized coreless super-Earths, which would not see any mantle oxidation from this mechanism. Since we consider multiple redox buffers for all planets, our analysis does not exclude the possibility of coreless super-Earths and the idea that Mars-sized planets may generally have more reduced mantles^27^. Figure 1 (middle panel) shows that the $\text {CO}_3^{2-}$ partitioning in the melt is suppressed in the reducing scenarios causing an enrichment in $\text {H}_{2}\text {O}$ in the rising melt and favoring an outgassing of $\text {H}_{2}\text {O}$ or $\text {H}_{2}$ over carbon-containing species. When the melt reaches the surface, the gas chemical speciation governs the partial pressure of the different outgassed volatile species (Fig. 1, bottom panel) and the atmospheric composition. Figure 4 shows the variation of the gas species due to different melt oxidation states. Depending on the initial volatile composition, $\text {CO}$, $\text {H}_{2}$ and $\text {H}_{2}\text {O}$ dominate the outgassing for the reduced mantle case whereas $\text {H}_{2}\text {O}$ and $\text {CO}_{2}$ dominate for the oxidised case. Assessing the melt fluxes and the volatile outgassing, we analysed atmospheric growth and evolution over time. Masses from 2 to 4 Earth masses are the most efficient in depleting the mantle of volatiles and outgassing large volumes of gas. As shown in Fig. 4, the reducing scenarios produce a larger atmospheric thickness compared to the oxidised cases. This is due to the different atmospheric composition between the two analysed cases. In the reducing case, the smaller molecular weights of $\text {CO}$, $\text {H}_{2}\text {O}$ and $\text {H}_{2}$ compared to $\text {CO}_{2}$ favour atmospheres which are less dense and which have a larger atmospheric scale height.

Considering atmosphere evolution, one aspect that affects the final composition is hydrogen escape, which was treated in a simplified way in Fig. 5. In general, transport (via eddy or molecular diffusion) together with photochemistry of H-containing species from the surface to the lower boundary of the homopause can result in the loss of atomic hydrogen from the atmosphere to space. Diffusion-limited escape depends on the atmospheric species, the hydrogen mixing ratio and the scale height of the atmosphere (Eq. 27). The diffusion of hydrogen to space is limited by the rate of transport of molecules from the atmosphere below. On the other hand, the loss of hydrogen dominated atmospheres due to the host star can be limited by the XUV energy which is dependent upon several factors such as stellar age, rotation and activity^28^. For planets with strong hydrogen outgassing or/and Super-Earths which retain hydrogen envelopes, $\text {H}_{2}$ can modify climate by influencing the greenhouse affect^29^. Regarding the loss of hydrogen from early Earth’s atmosphere, a rapid steam collapse leading to the formation of a water ocean would likely result in enhanced hydrogen escape from the atmosphere due to the strong EUV radiation of the young Sun. Atmospheric sinks (e.g. erosion, condensation, carbonate formation) and chemical reactions in the atmosphere can change the redox state and composition of the atmosphere^30,31^, but a reduced interior with strong, long-lasting volcanic activity would be expected to replenish reduced gases to the atmosphere, which might lead to detectable signatures.

The forthcoming PLATO mission^32,33^ will observe planetary radii down to an accuracy of 3%. For an Earth-like (Venus-like) planet, making the (rather approximate) assumption that transit measurements will be made in the atmosphere above $\sim$ 70 km (200 km) depending on wavelength, suggests that the atmospheric contribution to the observed radius makes up $\sim$ 1.1 (3.3) percent overall. We showed that the redox state of the mantle is one of the main factors influencing the thickness of the atmosphere and its evolution, translating into a few percent change (all other things being equal) in the observed planetary radius. This range is indeed comparable with the detection accuracy of the PLATO mission. The interior redox state of rocky planets could therefore be constrained with PLATO data especially for the thicker, Venus-like terrestrial atmospheres orbiting quieter stars where atmospheric escape is kept to a modest level and where condensation of water is unlikely.

In conclusion, our simulations show that redox-dependent geophysical models can improve interpretation of observed atmospheric data and provide a first-order characterisation of the interior chemical state of rocky exoplanets. Future observations of the atmospheric composition could give further constraints on the interior. Knowledge about the reducing or oxidising state of an atmosphere can guide future selection of target candidates for follow-up missions to detect a potentially habitable or even inhabited planet.

## Methods

### Melting and volatile partitioning

For investigating the transfer of volatiles from the interior to the atmosphere we use a 2D thermal evolution model to simulate mantle convection and melt production over time. In order to cover a wide range of different possible exoplanets, according to^16,18^ we vary the initial conditions of the system considering different planet masses (from 1 to 8 Earth masses), Mg/Si and Fe/Si ratios (0.5, 1 and 1.5 times solar values), distribution of iron between mantle and core (going from small cores with a high mantle iron content to the largest possible iron cores), the initial lithosphere thickness (50–100 km), initial upper mantle temperature (from 1600 to 2000 K beneath an initial lithosphere), temperature difference between core and mantle, initial amount of radiogenic heat sources (from 0.5 to 1.5 times Earth values), different mantle rheology and a wet compared to a dry mantle. For more details on the parameter cases we refer the reader to^18^. Melting occurs locally if the mantle temperature rises above the solidus melting temperature^34,35^ and we assume that 10%^36^ of the melt is immediately extracted to the surface and contributes to outgassing. The amount of melt depends on the rock composition that affects the solidus temperature, the internal thermal state and the size of the planet. The melting temperature is affected by variable initial iron and volatile content of the mantle^16,18^. The internal thermal state (i.e. the initial mantle temperature and the quantity of radioactivity heat sources) regulates the production of melt. Melting leads to the mantle being depleted in volatiles.

Our mantle and melt model is based on previous outgassing studies^16,18^ conducted with the convection code Crust, Habitability, and Interior Code (CHIC). CHIC is a 2D convection code which simulates the mantle convection in a stagnant lid tectonic regime solving the conservation equations of mass, momentum and energy in the rocky mantle^16^. We use a regional 2D spherical annulus geometry^37^ with the mantle being divided into cells with height of 25 km each. Pressure dependent parameters such as mineral-dependent density, thermal expansion coefficient and heat capacity for an adiabatic temperature profile are employed as in^18^. We model a compressible mantle by employing the truncated anelastic liquid approximation (TALA). In^18^, we assumed for simplicity that the mantle is homogeneously mixed at all times, and that its hydrogen and carbon are continuously depleted upon melting. However, in the present study, we a posteriori employ a redox-dependent partitioning of carbonates into the melt based on^38–40^, and consider partitioning of water into the melt based on^35^. We apply a batch melting model, based on our assumption that the melt is in equilibrium with the source rock before it rises to the surface. The partitioning of volatiles from the rock to the melt depends on the mantle-averaged melt fraction *F* and partition coefficient $D_{\text {H}_{2}\text {O}}$ via,

1 $$\begin{aligned} X_{\text {H}_{2}\text {O}}^\text{melt} = \frac{X_{\text {H}_{2}\text {O}}^\text{rock}}{D_{\text {H}_{2}\text {O}}+F(1-D_{\text {H}_{2}\text {O}})}, \end{aligned}$$

where we take $D_{\text {H}_{2}\text {O}}$ to be constant and equal to 0.01 ^41^.

We assume that carbon is stored in the mantle in the form of graphite, and dissolves into the melt in the form of carbonate ions, $\text {CO}_3^{2-}$. The amount of carbonate present is directly linked to the oxygen fugacity, $f_{\text {O}_{2}}$^38,42^. The $\text {CO}_{2}$ abundance in the melt used for the gas speciation model can then be calculated as follows:

2 $$\begin{aligned} X_{\text {CO}_{2}}^\text{melt} = \left[ \frac{M_{\text {CO}_{2}}}{\mathrm{fwm}} \, X_{\text {CO}_3^{2-}}^\text{melt} \right] \; \Bigg / \; \left[ 1 - \left( 1-\frac{M_{\text {CO}_{2}}}{\mathrm{fwm}} \right) X_{\text {CO}_3^{2-}}^\text{melt}\right] , \end{aligned}$$

where $M_{\text {CO}_{2}}$ is the molar mass of carbon dioxide and fwm is the formula weight of the melt where the number of atoms considered in the lattice unit are expressed per oxygen atom. In this way we assume fwm = 36.594 based on the 1921 Kilauea tholeiitic basalt following the approach of^40^.

The amount of carbonates dissolved in the melt can be calculated from equilibrium constants $K_{1}$ and $K_{2}$:

$$\begin{aligned} X_{\text {CO}_3^{2-}}^\text{melt}= & {} \frac{K_{1}\, K_{2} \, f_{\text {O}_{2}}}{1+K_{1} \, K_{2} \, f_{\text {O}_{2}}} \\ \log _{10} K_{1}= & {} 40.07639 - 2.53932\times 10^{-2} \; T \\&+ 5.27096\times 10^{-6} \; T^2 + 0.0267 \; \frac{p-1}{T}\\ \log _{10} K_{2}= & {} -6.24763 - \frac{282.56}{T} - 0.119242 \; \frac{p-1000}{T} \end{aligned}$$

for temperature, *T*, in K and pressure, *p*, in bar, and where $f_{\text {O}_{2}}$ is calculated based on different assumed redox buffers as described in Sect. 4.2.

For a more realistic treatment of volatile depletion in the mantle upon melting, we divide the mantle into two different volatile reservoirs, namely the upper and lower mantle respectively. We assume here that the mineral phase transition for ringwoodite to perovskite which separates the mantle into these two reservoirs takes place at 23 GPa (hence not accounting for any thermal effects on the transition pressure). The mantle pressure profile is directly obtained from the interior structure model that also gives us depth-dependent profiles for material parameters such as density and thermal expansion coefficient depending on our input mantle composition. The assumed initial volatile content (see Table 1) is homogeneously applied to the entire mantle. Melting depletes the upper mantle due to partitioning of volatiles into the melt (Eqs. 1, 2).

Mixing between upper and lower mantle depends on the convective velocity *v* of the mantle. While our convection simulation gives us information on the local convective strength, a global measure for the efficiency of mixing can be directly obtained from a common scaling law^38^ linking the average mantle convective velocity with the composition-dependent Rayleigh number *Ra*, which is a non-dimensional indicator of the convection efficiency:

3 $$\begin{aligned} v= & {} 2\cdot 10^{-12} \left( \frac{Ra}{450}\right) ^{(1/3)} \text{(m/s)}, \end{aligned}$$

4 $$\begin{aligned} Ra= & {} \frac{\rho g \alpha \Delta T D^3}{\kappa \eta} \end{aligned}$$

The Rayleigh number *Ra* depends on the density $\rho$, gravitational acceleration *g*, thermal expansion coefficient $\alpha$, mantle thickness *D*, thermal diffusivity $\kappa$ and mantle viscosity $\eta$. $\Delta T$ is the temperature contrast across the mantle. Following common mantle convection parameterizations (which in our study are based on^38^), the volume of mantle material transported from the lower into the upper mantle can then be estimated via:

5 $$\begin{aligned} V_\text{exch} = 0.5 v \ dt \ A_{tr}, \end{aligned}$$

where *dt* is the time step in seconds and $A_{tr}$ is the surface area of the boundary between upper and lower mantle. Note we do not take into account that the phase transition from upper to lower mantle can reduce convective material exchange; we therefore overestimate volatile outgassing during the earlier stages of planetary evolution.

The average volatile content of the upper mantle (‘um’) and lower mantle (‘lm’) is then calculated in each time step as follows:

6 $$X_{{{\text{H}}_{2} {\text{O,new}}}}^{{{\text{rock,um}}}} = X_{{{\text{H}}_{2} {\text{O}}}}^{{{\text{rock,um}}}} \frac{{V_{{{\text{um}}}} - V_{{{\text{exch}}}}}}{{V_{{{\text{um}}}}}} + X_{{{\text{H}}_{2} {\text{O}}}}^{{{\text{rock,lm}}}} \frac{{V_{{{\text{exch}}}}}}{{V_{{{\text{um}}}}}}$$

7 $$X_{{{\text{H}}_{2} {\text{O}},{\text{new}}}}^{{{\text{rock,lm}}}} = X_{{{\text{H}}_{2} {\text{O}}}}^{{{\text{rock,lm}}}} \frac{{V_{{{\text{lm}}}} - V_{{{\text{exch}}}}}}{{V_{{{\text{lm}}}}}} + X_{{{\text{H}}_{2} {\text{O}}}}^{{{\text{rock,um}}}} \frac{{V_{{{\text{exch}}}}}}{{V_{{{\text{lm}}}}}}$$

When melting occurs in the upper mantle, and if the melt is buoyant^43,44^, we assume that it is transported instantaneously upwards to the surface. Based on modern Earth average values for continental crust^36^, we expect that about $X_\text{extr}$ = 10% of the melt reaches the planet surface as extrusive melt and contributes to degassing into the atmosphere and we set this value as input in our model. In the discussion section, we further discuss possible contributions from intrusive melt pockets in the crust.

### Gas speciation model

To simulate the gas chemical speciation at different redox states we consider the Iron–Wustite (IW) and the Quartz–Fayalite–Magnetite (QFM) petrological mineral buffers. A mineral buffer is commonly employed in experimental petrology to keep constant the oxygen fugacity level in a reaction and to reproduce an oxidised or reduced state. By simulating the behaviour of mineral buffers, we calculate the oxygen fugacity of the system at different pressures and temperatures. For simulating a reducing case we simulate the QFM buffer while for the oxidising scenario we reproduce a redox state associated to the IW buffer. Normally, one would expect the oxidation state to change depending on the mantle composition, the proportion of FeO to ${\hbox {Fe}_3\hbox {O}_4}$, and the degassing of reduced species^4,45,46^. For simplicity, we hold oxidation states to constant values corresponding to IW and QFM mineralogical buffers.

Volatile chemical speciation in the C–O–H system is calculated via the “Equilibrium and mass balance method”^39,47–50^ for a wide range of pressures, temperatures and oxygen fugacities. Since the oxidation state of the melt strongly affects the chemical speciation of the volatiles therein, a speciation model has to calculate the oxygen fugacity, $f_{\text {O}_{2}}$, at a given temperature and pressure.

We assume the following common petrological mineral buffers:

8 $$\begin{aligned}&2 \hbox {Fe} + \hbox {O}_2 \rightleftharpoons 2 \hbox {FeO}, \qquad \mathrm{(IW)} \end{aligned}$$

9 $$\begin{aligned}&3\hbox {Fe}_{2}\hbox {SiO}_{4} + \hbox {O}_2 \rightleftharpoons 2 \hbox {Fe}_{3}\hbox {O}_4 + 3\hbox {SiO}_{2}, \qquad \mathrm{(QFM)} \end{aligned}$$

**Fig. 7:**
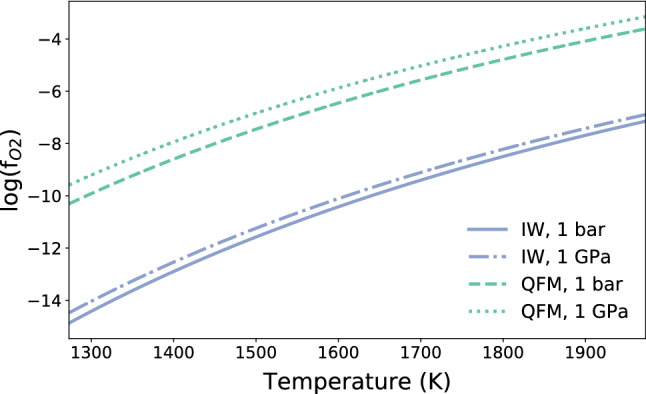
The variation of oxygen fugacity with melt temperature at different pressures for the IW and QFM redox buffers.

Each mineral buffer has a characteristic temperature-dependent $f_{\text {O}_{2}}$ curve^40^. Considering that the outgassing is simulated at the surface the pressure has only a negligible effect, as shown in Fig. 7. The calculated redox states are then used to estimate the partial pressures of each volatile ($\text {H}_{2}$, $\text {H}_{2}\text {O}$, $\text {CO}$ and $\text {CO}_{2}$) as a function of oxygen fugacity ($f_{\text {O}_{2}}$) following^23^. According to the approach of^49^, to estimate the volume of volatiles that remain in solution in the melt and the outgassed species we combine the gas-melt (solubility) with the gas–gas equilibria (degassing).

The gas-melt equilibria involved are:

10 $$\begin{aligned}&\hbox {H}_{2}\hbox {O}^{(fluid)} + \hbox {O}^{2- (\mathrm{melt})} \rightleftharpoons 2 \hbox {OH}^{-(\mathrm{melt})} \end{aligned}$$

11 $$\begin{aligned}&\hbox {CO}_2^{(\mathrm{fluid})} + \hbox {O}^{2- (melt)} \rightleftharpoons \hbox {CO}_3^{2- (\mathrm{melt})} \end{aligned}$$

12 $$\begin{aligned}&\hbox {H}_2^{(\mathrm{fluid})} \rightleftharpoons \hbox {H}_2^{(\mathrm{melt})} \end{aligned}$$

Considering the atmospheric pressure at the surface as degassing pressure, equilibria (10) and (11) are simulated according to^51^ while we assume that all the $\text {H}_{2}$ and the $\text {CO}$ are outgassed because of their low solubilities in silicate melts^52–54^.

Hydrogen is distributed between $\text {H}_{2}$ and $\text {H}_{2}\text {O}$ via the following gas–gas equilibrium:

13 $$\begin{aligned} 2 \hbox {H}_2^{(\mathrm{fluid})} + \hbox {O}_2 \rightleftharpoons 2 \hbox {H}_2\hbox {O}^{(\mathrm{fluid})}. \end{aligned}$$

The equilibrium constant ($K_3$) for this reaction can be expressed:

14 $$\begin{aligned} K_3 = \exp \left( \frac{-\Delta _rG^0_3}{RT}\right) , \end{aligned}$$

where *R* is the universal gas constant (8.314 J K$^{-1}$ mol$^{-1}$), *T* denotes the temperature of the degassed material in Kelvin, and $\Delta _rG^0_3$ denotes the Gibbs free energy of the reaction in Eq. (13). The latter depends only on the Gibbs free energy of formation of water, $\Delta _fG^0_{\text {H}_{2}\text {O}}$, via

15 $$\begin{aligned} \Delta _rG^0_3 = 2\Delta _fG^0_{\text {H}_{2}\text {O}}. \end{aligned}$$

Knowing $K_3$ one can calculate the ratio of $\text {H}_{2}\text {O}$ to $\text {H}_{2}$ at equilibrium. This has a direct dependence on $f_{\text {O}_{2}}$:

16 $$\begin{aligned} \left( \frac{X_{\text {H}_{2}\text {O}}}{X_{\text {H}_{2}}}\right) ^2 = K_3 f_{\text {O}_{2}}, \end{aligned}$$

Carbon is distributed with the gas–gas equilibrium between $\text {CO}$ and $\text {CO}_{2}$ via:

17 $$\begin{aligned} \hbox {CO}^{(\mathrm{fluid})} + 1/2 \hbox {O}_2 \rightleftharpoons \hbox {CO}_2^{(\mathrm{fluid})}, \end{aligned}$$

whose relative pressures are similarly set by the corresponding equilibrium constant,

18 $$\begin{aligned} K_4 = \exp \left( \frac{-\Delta _rG^0_4}{RT}\right) = \frac{X_{\text {CO}_{2}}}{X_{\text {CO}}}\frac{1}{f_{\text {O}_{2}}^{1/2}}, \end{aligned}$$

where

19 $$\begin{aligned} \Delta _rG^0_4 = \Delta _fG^0_{\text {CO}_{2}} - \Delta _fG^0_{\text {CO}}. \end{aligned}$$

The values of $\Delta _fG^0$ for each species have been determined empirically and can be found in standard thermodynamic tables from the literature^55^ (Table 2). This enables the calculation of volatile chemical speciation at equilibrium from the temperature, pressure and oxidation state of the system.

**Table 2:** Standard Gibbs free energies of formation used in this work are calculated via the gas reactions from^48^, valid for 1 bar pressure and the temperature (*T*) range 298–2500 K.

	*a*	*b*	*c*
**∆**_***f***_G^**0**^** = ** ***a* +** ***bT*** **log** ***T* +** ***cT***			
2 C + $\text {O}_{2}$ = 2 $\text {CO}$	− 214104.0	25.2183	− 262.1545
C + $\text {O}_{2}$ = $\text {CO}_{2}$	− 392647.0	4.5855	− 16.9762
2 $\text {H}_{2}$+ $\text {O}_{2}$= 2 $\text {H}_{2}\text {O}$	− 483095.0	25.3687	21.9563

Carbon is considered to exist in the form of graphite in our analysis.

We do not consider atmospheric climate, photochemistry or convection. Processes such as the hydrological cycle, surface-atmosphere exchange and atmospheric escape are the subject of future work. We set the degassing temperature to the liquidus melting temperature, which is about 2000 K [e.g., ^35^] and the surface pressure as outgassing pressure. Furthermore, we do not include O$_2$ since the O$_2$ partial pressure in the mantle is at least five orders of magnitude lower ($10^{-5}$ bar) compared to other volatile species^39,47^. Similarly, $\text {CH}_{4}$ concentrations in a high-temperature and low pressure magmatic context are insignificant, if present at all^56,57^. As reported by^27,58,59^, methane starts to be present at redox conditions below the IW buffer and at very high pressure at depth within the lithosphere. Therefore, in our simulations we exclude the methane because we do not reproduce reducing states below the IW buffer and we simulate the degassing at the surface, where the atmospheric pressure never reaches values comparable to the lithostatic pressures.

### Volatile outgassing and atmospheric height

We obtain melt fluxes over time from our mantle convection code, together with the gas fractions of the species $\text {CO}$, $\text {CO}_{2}$, $\text {H}_{2}$, and $\text {H}_{2}\text {O}$ depending on volatile content, temperature, and redox state of the melt.

The masses of the various atmospheric species which accumulate over time *k* are calculated via:

$$\begin{aligned} M_\text{atm}^{\text {H}_{2}\text {O}}= & {} X_\text{extr} \sum \limits _{k=2}^{n} X_{\text {H}_{2}\text {O}}^\text{melt}(t_k) F_k V_\text{mantle} \rho _\text{mantle} \frac{X_{\text {H}_{2}\text {O}}}{X_{\text {H}_{2}}+X_{\text {H}_{2}\text {O}}}\\ M_\text{atm}^{\text {H}_{2}}= & {} X_\text{extr} \sum \limits _{k=2}^{n} X_{\text {H}_{2}\text {O}}^\text{melt}(t_k) F_k V_\text{mantle} \rho _\text{mantle} \frac{X_{\text {H}_{2}}}{X_{\text {H}_{2}}+X_{\text {H}_{2}\text {O}}} \frac{M_{\text {H}_{2}}}{M_{\text {H}_{2}\text {O}}}\\ M_\text{atm}^{\text {CO}_{2}}= & {} X_\text{extr} \sum \limits _{k=2}^{n} X_{\text {CO}_{2}}^\text{melt}(t_k) F_k V_\text{mantle} \rho _\text{mantle} \frac{X_{\text {CO}_{2}}}{X_{\text {CO}}+X_{\text {CO}_{2}}}\\ M_\text{atm}^{\text {CO}}= & {} X_\text{extr} \sum \limits _{k=2}^{n} X_{\text {CO}_{2}}^\text{melt}(t_k) F_k V_\text{mantle} \rho _\text{mantle} \frac{X_{\text {CO}}}{X_{\text {CO}}+X_{\text {CO}_{2}}} \frac{M_{\text {CO}}}{M_{\text {CO}_{2}}} \end{aligned}$$

where $F_k$ is the average melt fraction at time $t_k$. $X_{\text {H}_{2}}$, $X_{\text {H}_{2}\text {O}}$, $X_{\text {CO}}$ and $X_{\text {CO}_{2}}$ are mole fractions determined as described in Sect. 4.2. $V_\text{mantle}$ is the volume of the mantle and $\rho _\text{mantle}$ is the average mantle density. $X_\text{extr}$ denotes the fraction of melt that we assume to reach the surface via extrusive volcanism, which we set here to 10%, which is in the range of observed values for Earth’s continental crust^36^.

To estimate atmospheric extent one has to relate the molecular weight of outgassed species $M_\text{atm}^{i}$ to their partial pressures $p_\text {s}^i$. The *total* pressure $p_\text {s}$ at a given point in the atmosphere is proportional to the *total* overhead weight of the atmospheric column which can be estimated via the hydrostatic relation assuming vertical motions are negligible via:

20 $$\begin{aligned} p_\text {s} = \frac{g}{A} M_\text {atm}, \end{aligned}$$

where *g* is the surface gravity and *A* the total surface area of the planet. The total pressure and mass are given given by:

21 $$\begin{aligned} p_\text {s} = \sum _i p_\text {s}^i \quad \text {and} \quad M_\text {atm} = \sum _i M_\text {atm}^i . \end{aligned}$$

Using the volume mixing ratio $x_i$, the partial pressure of a species *i* is determined by $p_\text {s}^i = x_i p_\text {s}$, while the corresponding atmospheric mass is determined via the mass mixing ratio $w_i$ according to: $M_\text {atm}^i = w_i M_\text {atm}$.

The relationship between these two mixing ratios—assuming an ideal gas—is given by:

22 $$\begin{aligned} w_i = \frac{\rho _i}{\rho} = \frac{m_i}{\bar{m}} \frac{n_i}{n} = \frac{m_i}{\bar{m}} \frac{p_\text {s}^i}{p_\text {s}} = \frac{m_i}{\bar{m}} x_i , \end{aligned}$$

where $\rho$ is the mass density, *n* the number density, $m_i$ the molecular weight of species *i*, and $\bar{m}$ the mean molecular weight, given by:

23 $$\begin{aligned} \bar{m} = \sum _i x_i m_i . \end{aligned}$$

Using this relation, Eq. (20) for a single species *i*, that is well-mixed throughout the atmosphere, is given by:

24 $$\begin{aligned} p_\text {s}^i = \frac{g}{A} \frac{\bar{m}}{m_i} M_\text {atm}^i . \end{aligned}$$

Consequently, the partial pressure of a given atmospheric species depends on both the number densities as well as the molecular masses of all species present. For a given column mass density, going from a composition of rather heavy molecules (e.g. $\text {CO}_{2}$ or $\text {H}_{2}\text {O}$) to lighter species (e.g. $\text {H}_{2}$) will reduce the total surface pressure and, hence, could strongly impact habitability. The effect is even stronger at larger surface gravities.

In our work, the atmospheric thickness ($\Delta R_p$) is defined from the planetary surface to the pressure $p_\text{min}$. To calculate the atmospheric thickness, given the accumulated amounts of outgassed species (e.g. $\text {H}_{2}\text {O}$, $\text {CO}_{2}$, $\text {H}_{2}$), we use a simple scale height model following^60^:

25 $$\begin{aligned} \Delta R_p = H \ln {\left( \frac{p_\text{tot}}{p_\text{min}}\right)}, \end{aligned}$$

where $p_\text{tot}$ is the total gas pressure. $p_\text{min}$ is the pressure at which the atmosphere becomes opaque that is where the chord optical depth becomes 0.6. $p_\text{min}$ is defined as:

26 $$\begin{aligned} p_\text{min} \approx \frac{g}{\kappa} \sqrt{\frac{H}{2\pi R_p}}. \end{aligned}$$

If we assume a mean opacity of $\kappa = 0.1$ cm$^2$ g$^{-1}$^61^ , then we obtain $p_\text{min} \approx 1$ mbar. Our main focus is the relevance for habitable planets. To this end, the pressure scale height *H* is calculated assuming a mean atmospheric temperature $T_\text{atm}$ of 288 K, which is the Earth’s average surface temperature:

27 $$\begin{aligned} H ={ \frac{R T_\text{atm}}{\bar{m} \cdot g}} \end{aligned}$$

where *g* is surface gravity, *R* is the universal gas constant (8.3144598 J mol$^{-1}$ K$^{-1}$), and $\bar{m}$ is again the mean molecular weight of the gas mixture.

## Supplementary information

Supplementary file1 (PDF 512 kb)
