# Supplementary material for: Mantle redox state drives outgassing chemistry and atmospheric composition of rocky planets
Source: Sci Rep. 2020 Jul 2;10:10907. doi: 10.1038/s41598-020-67751-7 (PMC7331660; doi:10.1038/s41598-020-67751-7)
Supplement: Supplementary file 1 — Supplementary file1 (PDF 512 kb) [file 41598_2020_67751_MOESM1_ESM.pdf]

SM Supplementary materials

SM.1 Passive outgassing and solubility effect

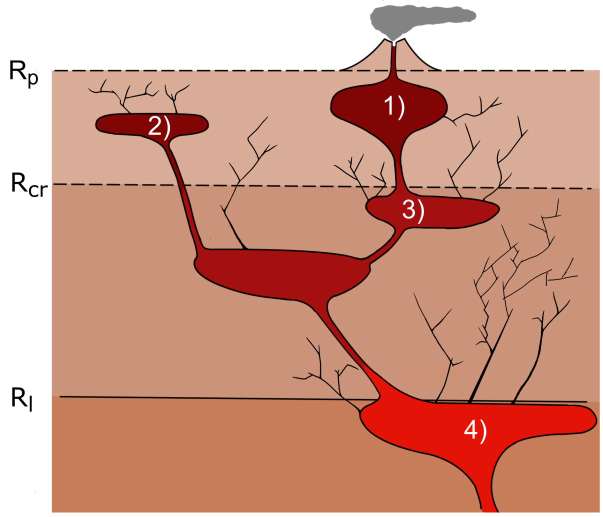

Figure 8: Magma chamber depth zones. 1) extrusive shallow volcanism, 2) intrusive crustal magmatism, 3) magmatic underplating, 4) chambers below the lithosphere. Rp: Planet radius, Rcr: Crust radius and Rl: Lithosphere radius. Through the illustrated cracks (black), outgassing from lower magma levels is becoming possible. In the analysed cases, we considere the outgassing contribution from the magma at the surface.

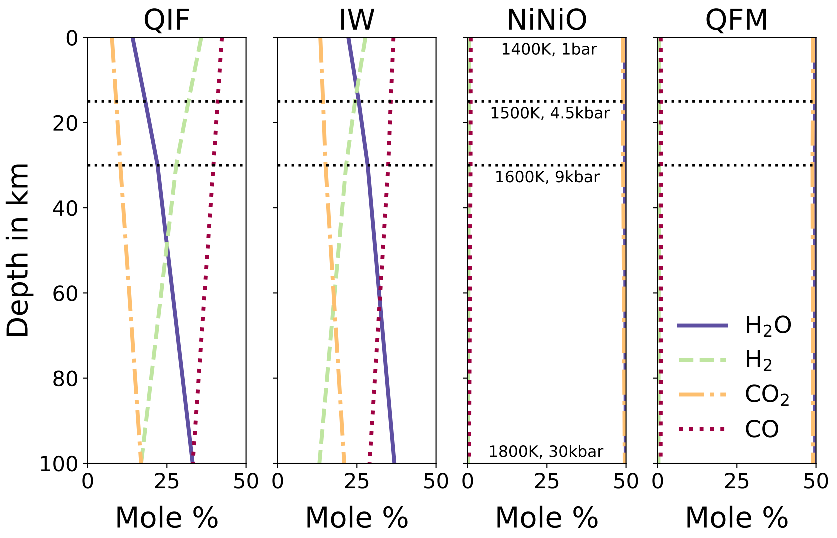

Figure 9: Depth profiles of the gas speciation variation depending on redox state, pressure and temperature for intrusive melt. We assume here a fixed volatile ratio of 50 mol% H_2O and 50 mol% CO_2 in the melt. Temperature and pressure values for cases 1) to 4) in Fig. 8 are shown in the NiNiO panel.

Depending upon the depth (hence pressure) at which the melt is produced, degassing could begin soon after magma formation. The total amount of outgassing is the combination of gases released both from deep magmatic emplacements (passive degassing) and from the melt extruded to the surface. Figure 8 shows magma chambers at different depths within or directly below the lithosphere and cracks and fissures where the gas ascends to the surface. The lithostatic pressure and the mantle redox state affect the volatile content in the melt and the final outgassing. Figure 9 illustrates how pressure influences gas species' amounts; the effect is stronger under

reducing conditions (QIF and IW mineral buffers). On increasing the pressure, the H_2O and CO_2 content increase with similar trends while the volume of CO and H_2 decrease. For oxidising states (NiNiO and QFM mineral buffers) the pressure effect on the speciation is negligible. In our example we assume as initial volatile content in the melt 50 mol % of both H_2O and CO_2. Global mean values based on modern Earth [36] suggest that 10% of the generated melt reaches the surface and directly contributes to the outgassing. Passive degassing on the Earth is larger than the extrusive component [36, 62]. Intrusive magma bodies emplaced at the bottom or within the lithosphere, continuously crystallise due to cooling. Since volatiles such as H_2O or CO_2 are incompatible in the crystal lattice of mafic minerals, they tend to concentrate in the melt. At a certain point, the concentration exceeds the solubility and a gas-phase forms [63, 64, 65, 66, 67, 62]. The gas is buoyant and ascends either through already-existing cracks and fissures or leads to overpressure which may itself result in cracks. Such intrusive degassing should strongly influence the amount as well as the composition of the outgassed volatiles, especially since the solubility of CO_2 is much lower compared to H_2O [e.g., 68, 64, 67, 62, 69], see figure 10.

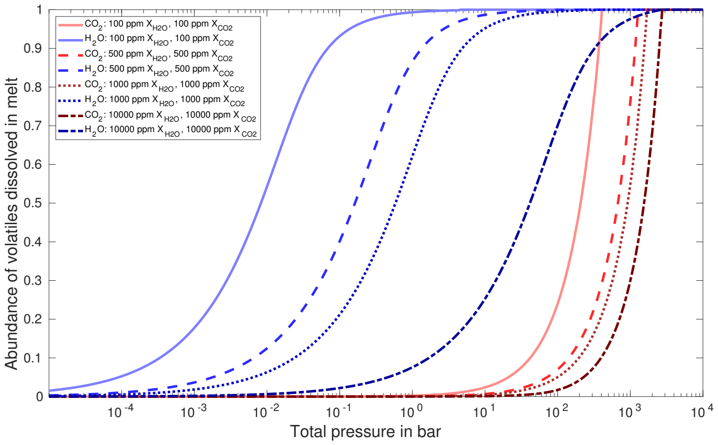

Figure 10: Solubility profiles for H_2O and CO_2 degassing depending on pressure for different volatile contents in the melt calculated after Iacono-Marziano [51].

Therefore, CO_2 degasses rather quickly, especially at low pressures, while H_2O is assumed to remain in the melt. Only at the very end of the crystallisation sequence, H_2O can be degassed at these low pressures, if the saturation limit is exceeded [70]. This tendency toward earlier CO_2 degassing should strongly affect both the amount as well as the composition of the outgassed volatiles. Since we performed the outgassing simulations at surface pressure, the contribution of passive degassing was negligible as the effect of crystallisation and the related oversaturation of the melt is not considered in our work. Even though, at higher pressures there should be a strong influence of passive degassing, especially since the pressure has a direct influence on degassed volatile species under reducing conditions (considered for early Earth), which is shown in figure 9. Depending on the redox buffer (QIF or IW), at very low pressures mainly CO and H_2 are degassed, while at higher pressures CO and H_2O degassing dominates. For oxidised conditions, the effect of pressure is negligible and for both buffers (NiNiO and QFM) only H_2O and CO are degassed. However, the effect of crystallization in magmatic intrusions is not considered here and should be a point for future studies.

SM.2 Volatile chemical speciation

In figures 11 and 12 we show how the melt oxidation state plays a crucial role for the gas chemical speciation and therefore for atmospheric evolution.

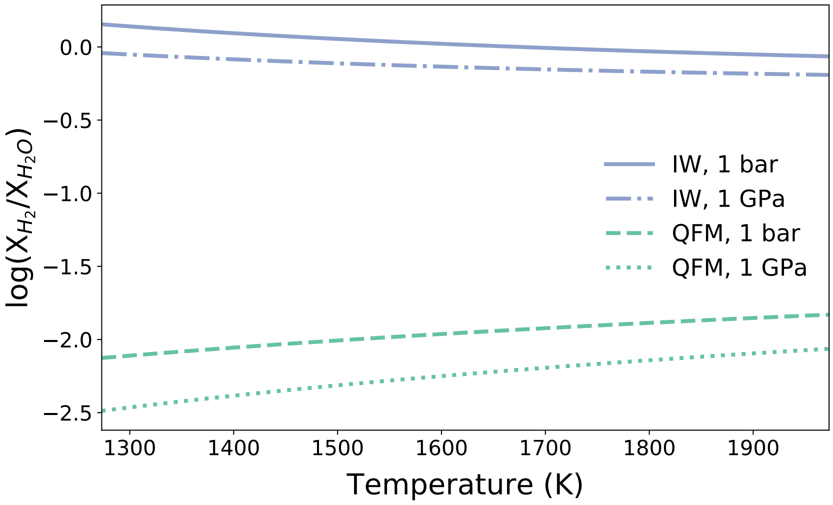

Figure 11: H_2-to-H_2O mole ratio for different temperatures and mineral redox buffers where QFM is the oxidising case and IW the reducing scenario. H_2-to-H_2O mole ratio for the IW buffer is described at 1 bar with the solid line and at 1 GPa (1 Gpa = 10000 bar) by the dashed and dotted line. H_2-to-H_2O mole ratio for the QFM buffer is represented at 1 bar with the dashed line an at 1 GPa by the dotted line.

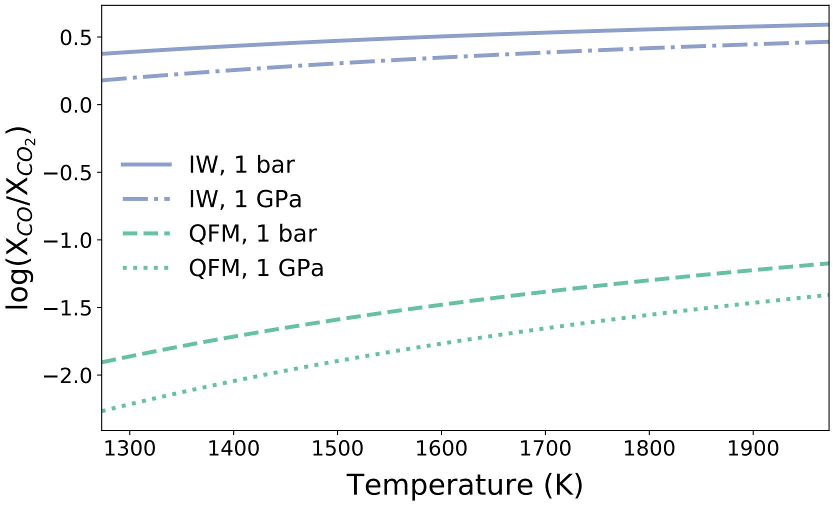

Figure 12: CO-to-CO_2 mole ratio for different temperatures and mineral redox buffers where QFM is the oxidising case and IW the reducing scenario. CO-to-CO_2 mole ratio for the IW buffer is described at 1 bar with the solid line and at 1 GPa (1 Gpa = 10000 bar) with the dashed and dotted line. CO-to-CO_2 mole ratio for the QFM buffer is represented at 1 bar by the dashed line an at 1 GPa by the dotted line.
